# Motor Dysfunction Questionnaire and Dopamine Transporter Imaging Composite Scale Improve Differentiating Dementia With Lewy Bodies From Alzheimer's Disease With Motor Dysfunction

**DOI:** 10.3389/fnagi.2021.709215

**Published:** 2021-08-11

**Authors:** Pai-Yi Chiu, Cheng-Yu Wei, Guang-Uei Hung, Shey-Lin Wu

**Affiliations:** ^1^Department of Neurology, Show Chwan Memorial Hospital, Changhua, Taiwan; ^2^Department of Nursing, College of Nursing and Health Sciences, Da-Yeh University, Changhua, Taiwan; ^3^Department of Neurology, Chang Bing Show Chwan Memorial Hospital, Changhua, Taiwan; ^4^Department of Nuclear Medicine, Chang Bing Show Chwan Memorial Hospital, Changhua, Taiwan; ^5^Department of Neurology, Changhua Christian Hospital, Changhua, Taiwan; ^6^Department of Biomedical Sciences, Da-Yeh University,Changhua, Taiwan

**Keywords:** Alzheimer's disease, dementia with Lewy bodies, non-dementia, motor dysfunction, striatal–background ratio

## Abstract

**Objective:** Characteristic parkinsonism is the major comorbidity of dementia with Lewy bodies (DLB). We aimed to differentiate DLB from Alzheimer's disease (AD) with motor dysfunction using a composite scale with a characteristic motor dysfunction questionnaire (MDQ) and dopamine transporter (DAT) imaging. It could help detect DLB easily in healthcare settings without movement disorder specialists.

**Methods:** This is a two-phase study. In the design phase, seven questions were selected and composed of a novel MDQ. In the test phase, all participants with DLB, AD, or non-dementia (ND) control completed dementia and parkinsonism survey, the novel designed questionnaire, DAT imaging, and composite scales of MDQ and DAT. The cutoff scores of the MDQ, semiquantitative analysis of the striatal–background ratio (SBR) and visual rating of DAT, and the composite scale of MDQ and DAT for discriminating DLB from AD or ND were derived and compared.

**Results:** A total of 277 participants were included in this study (126 with DLB, 86 with AD, and 65 with ND). Compared with the AD or ND groups, the DLB group showed a significantly higher frequency in all seven items in the MDQ and a significantly lower SBR. For discrimination of DLB from non-DLB with MDQ, SBR, and composite scale, the cutoff scores of 3/2, 1.37/1.38, and 6/5 were suggested for the diagnosis of DLB with the sensitivities/specificities of 0.91/0.72, 0.91/0.80, and 0.87/0.93, respectively. The composite scale significantly improved the accuracy of discrimination compared with either the MDQ or SBR.

**Conclusion:** This study showed that the novel designed simple questionnaire was a practical screening tool and had similar power to DAT scanning to detect DLB. The questionnaire can be applied in clinical practice and population studies for screening DLB. In addition, the composite scale of MDQ and DAT imaging further improved the diagnostic accuracy, indicating the superiority of the dual-model diagnostic tool.

## Introduction

Dopamine transporter (DAT) imaging is an indicative biomarker for diagnosing dementia with Lewy bodies (DLB). A recent systematic analysis showed that the sensitivity and specificity for the differentiation of DLB from other brain disorders were 0.86 and 0.81 and 0.93 and 0.75 for visual and semiquantitative assessments of DAT-Single Photon Emission Computed Tomography (DAT-SPECT), respectively (Nihashi et al., 2020). However, there is still a lack of tracers that target α-synuclein, and DAT has become the most important biomarker for the diagnosis of DLB.

Clinical diagnosis of DLB is mainly based on consensus criteria, and the core clinical features help detect and differentiate DLB from other dementia syndromes. Core clinical features include fluctuations of cognition, characteristic visual hallucinations (VH), rapid eye movement (REM) sleep behavior disorder (RBD), and spontaneous parkinsonism. For the clinical detection of DLB, Galvin (2015) provided a simple risk score composed of 10 questions associated with core/supportive clinical features.

Parkinsonism is a cardinal feature for the diagnosis of Parkinson's disease (PD) (Emre et al., 2007; Postuma et al., 2015) and a core clinical feature for the diagnosis of DLB (McKeith et al., 2017). However, PD is also comorbid with many other neurological and systemic disorders. The prevalence rate of the coexistence of parkinsonism and AD may be higher than previously recognized (Lopez et al., 1997; Sasaki, 2018). Clinical and differential diagnosis of DLB can only be made accurately by acquiring a detailed clinical history and performing neurological examinations, neuroimaging, or other laboratory studies. The characteristics of parkinsonism associated with DLB are different from motor dysfunction due to other common disease entities, such as essential tremor (ET), skeletal disorders, cardiovascular diseases, cerebrovascular diseases, and frailty in older adults. In particular, the characteristics of parkinsonism associated with DLB are unfamiliar and difficult for researchers or clinicians without well-trained skills in taking neurological history and/or performing the neurological examination. Therefore, several clinical and community-based screening questionnaires for PD were designed and studied with a sensitivity of 48–100% and a specificity of 22–100% (Tanner et al., 1990; Mutch et al., 1991; Chang et al., 1996; The Italian Longitudinal Study on Aging Working Group, 1997; Chan et al., 2000; Dahodwala et al., 2012). The screening efficacies of these scales on clinical or community populations vary (Dahodwala et al., 2012). However, none of these scales addressed the discrimination of motor dysfunction between DLB and other dementia disorders, although DLB is the second most common degenerative dementia (McKeith et al., 2005, 2017; Zaccai et al., 2005), and the clinical presentations of DLB and Alzheimer's disease (AD) dementia are easily confused.

Based on clinical experience and the previous study on the diagnosis of parkinsonism, we also found some discrepancies between the characteristic motor symptoms described by caregivers and the performance of patients examined by physicians (Lin et al., 2018). To narrow down the differences, this study aims to compare the different presentations of parkinsonism to those with AD as observed by the caregivers of patients with DLB, and therefore, to design a simple motor dysfunction questionnaire (MDQ). The questionnaire was constructed with clinically frequent questions or usual complaints of characteristic motor symptoms obtained from caregivers or patients in the clinics or bedsides and modified according to the clinical diagnostic criteria for PD dementia (PDD) or DLB. In addition, we intended to validate the newly designed informant-based motor dysfunction screening tool by testing it among a registered-based population with a diagnosis objectively proven by DAT imaging. Furthermore, during the consecutive data collection, the embedded auto-judgment program in the questionnaire will continue to revise the weighting of each question using machine learning techniques to improve the diagnostic ability.

## Methods

### Participants

This was a two-phase study to design and test the MDQ embedded in the History-based Artificial Intelligence Clinical Dementia Diagnostic System (HAICDDS), which is currently used to register patients with dementia or motor dysfunction in the Show Chwan Healthcare System (Lin et al., 2018; Chiu et al., 2019a,b; Wang et al., 2020; Zhu et al., 2020). Before beginning the project, 30 patients with their caregivers were tested by neuropsychologists from three centers, and the reproducibility was studied using the interrater reliability analysis. Then, the coefficient was calculated to estimate the reliability of the newly developed questionnaire. After that, the baseline and follow-up data of participants were continuously collected, and the embedded diagnostic system was modified with machine learning techniques to improve the diagnostic accuracy and efficiency.

In the design phase (2014–2016), we retrospectively analyzed 13 motor-associated questions, including resting tremor, action tremor, bradykinesia, rigidity, postural instability, monotonic and hypotonic speech, jerk, impaired fine motor movement, restlessness, gait or truncal deviation, dystonic movement, asymmetric onset, and repeated falls in the early stage. These questions were selected based on the characteristic PD/DLB motor symptoms suggested by the criteria (Emre et al., 2007). Along with other common motor symptoms observed in patients with brain disorders, the original 13 questions were compared between the PD/DLB and AD groups. The first seven questions with the highest odds ratios (ORs) for the discrimination of PD/DLB from non-PD/DLB were selected to compose the MDQ (HAI-MDQ) (Supplementary Table 1).

In the test phase (2017–2020), the participants with DLB or AD who registered in the HAICDDS database with at least one DAT imaging were analyzed and compared for their HAI-MDQ, DAT imaging, and demographic, clinical, neuropsychological, and neuroimaging characteristics. The cutoff scores for HAI-MDQ and the striatal–background ratio (SBR) of DAT were derived. Composite scores of HAI-MDQ and SBR were calculated using the total HAI-MDQ score plus abnormal DAT (DATabN) by either visual rating (VR) or SBR. The weighting of DATabN by either VR or SBR was given as the same as the cutoff score for diagnosing DLB in HAI-MDQ based on a presumed equal diagnostic power of clinical and imaging tools.

### Diagnostic Procedures

The diagnosis of DLB was made according to the revised consensus criteria for probable DLB developed by the fourth report of the DLB consortium in 2017 (McKeith et al., 2017). Patients with AD were diagnosed according to the criteria for probable AD with dementia developed by the National Institute on Aging and Alzheimer's Association (NIA-AA) 2011 criteria (McKhann et al., 2011).

Neuropsychological tests, including cognitive and daily function, were assessed using the Montreal Cognitive Assessment (MoCA) (Chen et al., 2016) and instrumental activities of daily living (IADL) scales (Lawton and Brody, 1969). The tests for all patients were performed by trained neuropsychologists. The clinical features of DLB, including REM, RBD, VH, and cognitive fluctuations, were assessed by neurologists using a structured interview. Motor signs of all participants were assessed by neurologists using the motor subscale of the Unified Parkinson's Disease Rating Scale (UPDRS-m) (Ballard et al., 1997). The motor symptoms of each participant were assessed using the HAI-MDQ. In performing HAI-MDQ, the caregivers of the participants were interviewed by a well-trained neuropsychologist. They were requested to complete the whole HAICDDS questionnaire, including the 13-item motor questionnaire (the original Chinese version of the questionnaire with a tentative English translation is shown in Supplementary Table 1). DATabN derived from Tc99m TRODAT-1 imaging by VR was assessed by two nuclear medicine physicians using interrater reliability tests. Only participants with at least one cerebral structure imaging (CT or MRI) and Tc99m TRODAT-1 imaging were analyzed.

### Statistics

The Chinese version of SPSS 22.0 software for Windows (IBM, SPSS Inc., Chicago) was used for statistical analyses. For the composition of the MDQ, the chi-square test for each question in the HAI-MDQ was compared between the DLB and non-DLB groups. Demographic data, including sex, RBD, VH, cognitive fluctuation, DATabN, SBR, UPDRS-m, levodopa equivalent dose (LED), and neuropsychological tests, including Clinical Dementia Rating (CDR), IADL, MoCA, HAI-MDQ, and the sum of scores of the Neuropsychiatric Inventory (NPI-sum) (Ballard et al., 1997), were summarized. The cutoff scores of the HAI-MDQ and SBR to differentiate DLB from non-DLB were derived. To determine the cutoff scores and maximize both sensitivity and specificity, Youden's index was applied. A composite score of the HAI-MDQ and positive SBR were summed with a total score of 7.0, and the cutoff score was also derived and compared. ORs for each variable adjusted for age and disease severity (sum of boxes of the Clinical Dementia Rating scale, CDR-SB) were compared between the DLB and non-dementia (ND) groups, the DLB and AD groups, or HAI-MDQ+ and the HAI-MDQ–groups.

## Results

In the design phase, 253 participants with PD/DLB and 491 with non-PD/DLB were analyzed and compared for the 13 candidate symptoms for the composition of the HAI-MDQ. All 13 symptoms were much higher in the PD/DLB group than those in the non-PD/DLB group (all *p* < 0.001). Therefore, seven questions (i.e., MD01, MD02, MD03, MD04, MD05, MD07, and MD08) with the highest ORs were selected to compose the HAI-MDQ (Supplementary Table 1).

**Figure 1:**
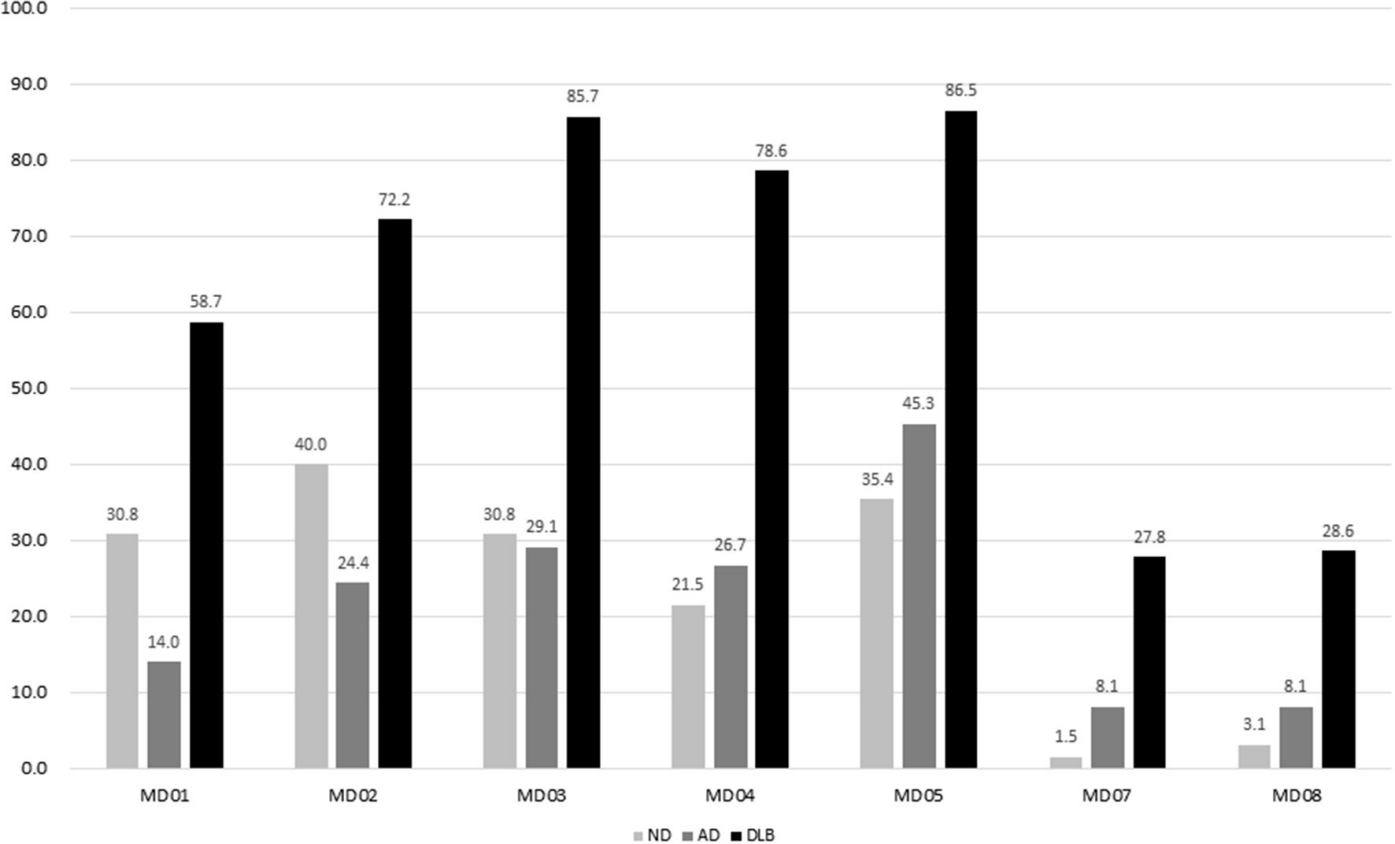
Prevalence of each item in MDQ among different diagnostic groups. MDQ, motor dysfunction questionnaire.

In the test phase, a total of 277 participants with complaints of motor dysfunction were referred for this study, including 65 participants with ND, 86 participants with AD, and 126 participants with DLB. The duration of motor dysfunction among patients with ND (2.0 ± 4.3), AD (1.0 ± 1.7), and DLB (2.3 ± 2.7) was significantly different (*p* = 0.010). The duration of dementia in patients with AD (2.8 ± 1.0) and DLB (2.5 ± 2.8) was not different. The frequencies of DATabN determined using VR by nuclear medicine physicians were significantly higher in the DLB group (92.1%) than those in the AD (30.2%) or ND (9.2%) groups. The prevalence of each item among the different diagnostic groups in the test phase is shown in Figure 1.

The selected items were equally weighed; therefore, the total HAI-MDQ score was 7.0. The comparison of the demographic data among the DLB, ND, and AD groups revealed significantly higher HAI-MDQ total score, UPDRS-m, LED, and lower SBR (all *p* < 0.001). The DLB non-motor features, including DATabN, RBD, cognitive fluctuations, and VH (all *p* < 0.005), were also significantly higher in the DLB group than those in the other groups (Table 1).

**Table 1:** Comparison of demographic data among the ND (*N* = 65), AD (*N* = 86), and DLB (*N* = 126) groups.

	**ND, mean (SD)**	**AD, mean (SD)**	**DLB, mean (SD)**	***p***	***Post-hoc*/paired comparison**
Age, year	75.1 (6.3)	75.3 (10.5)	76.9 (7.6)	NS	ND = AD = DLB
Female, N (%)	40 (61.5)	53 (61.6)	71 (56.3)	0.12	
Education, year	6.4 (4.4)	4.5 (4.4)	4.9 (9.7)	NS	ND = AD = DLB
Disease duration					
Dementia, year	-	2.8 (2.5)	2.5 (2.8)	NS	AD = DLB
Motor, year	2.0 (4.3)	1.0 (1.7)	2.3 (2.7)	0.010	ND = AD; ND = DLB; AD < DLB
CDR-SB	1.8 (0.8)	5.5 (3.9)	7.7 (4.4)	<0.001	ND < AD < DLB
CASI	76.4 (11.6)	50.1 (22.7)	47.3 (22.7)	<0.001	ND > AD = DLB
MoCA	17.5 (5.8)	9.9 (6.4)	8.4 (5.9)	<0.001	ND > AD = DLB
NPI-sum	5.5 (8.8)	9.8 (11.9)	13.7 (11.0)	<0.001	ND < AD < DLB
UPDRS-m	14.9 (9.9)	17.1 (14.3)	35.4 (19.3)	<0.001	ND = AD < DLB
LED	98.7 (136.9)	52.2 (117.3)	205.4 (202.3)	<0.001	ND = AD < DLB
MDQ	1.6 (1.6)	1.6 (1.5)	4.4 (1.5)	<0.001	ND = AD < DLB
DATabN, N (%)	6 (9.2)	26 (30.2)	116 (92.1)	<0.001	ND < AD < DLB
RBD, N (%)	10 (15.4)	10 (11.6)	67 (53.2)	<0.001	ND < AD < DLB
Fluctuation, N (%)	2 (3.1)	14 (16.3)	87 (69.0)	<0.001	ND < AD < DLB
VH, N (%)	0 (0.0)	11 (12.8)	52 (41.3)	<0.001	ND < AD < DLB

*ND, non-dementia control; AD, Alzheimer's disease; N, number of participants; DLB, dementia with Lewy bodies; NS, non-significance; CDR-SB, Sum of Boxes of the Clinical Dementia Rating scale; CASI, Cognitive Abilities Screening Instrument; MoCA, Montreal Cognitive Assessment; NPI-sum, sum score of neuropsychiatric inventory; UPDRS-m, the motor score of the Unified Parkinson's Disease Rating Scale; LED, levodopa equivalent dose; SBR, striatal–background ratio of dopamine transporter imaging; MDQ, motor dysfunction questionnaire in the History-based Artificial Intelligence Clinical Dementia Diagnostic System; DATabN, abnormal dopamine transporter imaging by VR; RBD, REM sleep behavior disorder; Fluctuation, fluctuation of cognition; VH, visual hallucinations*.

Among the participants with DLB, at least three symptoms of HAI-MDQ were reported in 91.2% of the DLB group. These symptoms were reported to be much lower in the non-DLB groups (30.8% for ND and 23.3% for AD). Therefore, a cutoff score of 3/2 for the total HAI-MDQ score was suggested for the screening of motor dysfunction due to DLB vs. non-DLB with a sensitivity of 0.91, a specificity of 0.72, and an area under the curve (AUC) of 0.89. A cutoff score of 1.37/1.38 for SBR in DAT imaging was derived with a sensitivity of 0.91, a specificity of 0.80, and an AUC of 0.90. Two types of composite scores were derived from a further combination of the questionnaire and DAT imaging. First, the composite score was combined with a total HAI-MDQ plus SBR (MDQSBR); if SBR <1.38, the weighing of SBR was scored as 3, which is the same as the cutoff score for DLB in HAI-MDQ; on the contrary, if SBR ≥ 1.38, the weighing of SBR was scored as 0. Second, the composite score was combined with the total HAI-MDQ plus VR of DAT (MDQVR) by a nuclear medicine physician. If the rating was abnormal, the weighing of VR was 3, which is the same as the weighing of SBR and the cutoff score of the abnormal MDQ. In contrast, if VR is negative, the score is 0. A cutoff score of 6/5 of the composite score of MDQSBR was derived for discriminating DLB from non-DLB with a satisfactory sensitivity, specificity, positive predictive value, negative predictive value, and AUC. The AUCs discriminating DLB from non-DLB in HAI-MDQ, SBR, and composite scores were 0.94, 0.89, and 0.96, respectively (Table 2).

**Table 2:** Comparison of SEN, SPEC, PPV, NPV, and AUC with 95% CI among the DLB vs. ND, DLB vs. AD, and DLB vs. non-DLB groups using a cutoff score of 3/2 for the HAI-MDQ, a cutoff score of 1.37/1.38 for the SBR, and a cutoff score of 6/5 for the combination of the MDQVR or MDQSBR.

	**SEN**	**SPEC**	**PPV**	**NPV**	**AUC (95% CI)**
HAI-MDQ
DLB vs. ND	0.91	0.68	0.85	0.80	0.87 (0.84–0.94)
DLB vs. AD	0.91	0.76	0.85	0.86	0.89 (0.84–0.94)
DLB vs. Non-DLB	0.91	0.72	0.73	0.91	0.89 (0.85–0.93)
SBR
DLB vs. ND	0.91	0.89	0.97	0.84	0.93 (0.89–0.97)
DLB vs. AD	0.91	0.72	0.83	0.85	0.93 (0.89–0.97)
DLB vs. Non-DLB	0.91	0.80	0.79	0.92	0.90 (0.86–0.94)
MDQVR
DLB vs. ND	0.87	0.97	0.98	0.79	0.98 (0.96–1.00)
DLB vs. AD	0.87	0.91	0.93	0.82	0.95 (0.93–0.98)
DLB vs. Non-DLB	0.87	0.93	0.92	0.89	0.96 (0.94–0.98)
MDQSBR
DLB vs. ND	0.86	0.97	0.98	0.78	0.98 (0.96–1.00)
DLB vs. AD	0.86	0.90	0.92	0.81	0.95 (0.92–0.98)
DLB vs. Non-DLB	0.86	0.93	0.91	0.89	0.96 (0.94–0.98)

*SEN, sensitivity; SPEC, specificity; PPV, positive predictive value; NPV, negative predictive value; AUC, area under the curve; DLB, Lewy body dementia; ND, non-dementia control; AD, Alzheimer's disease; SBR, striatal–background ratio of dopamine transporter imaging; HAI-MDQ, motor dysfunction questionnaire in the History-based Artificial Intelligence Clinical Dementia Diagnostic System; MDQVR, the composite scale of MDQ and DAT VR scale; MDQSBR, the composite scale of MDQ and SBR*.

**Figure 2:**
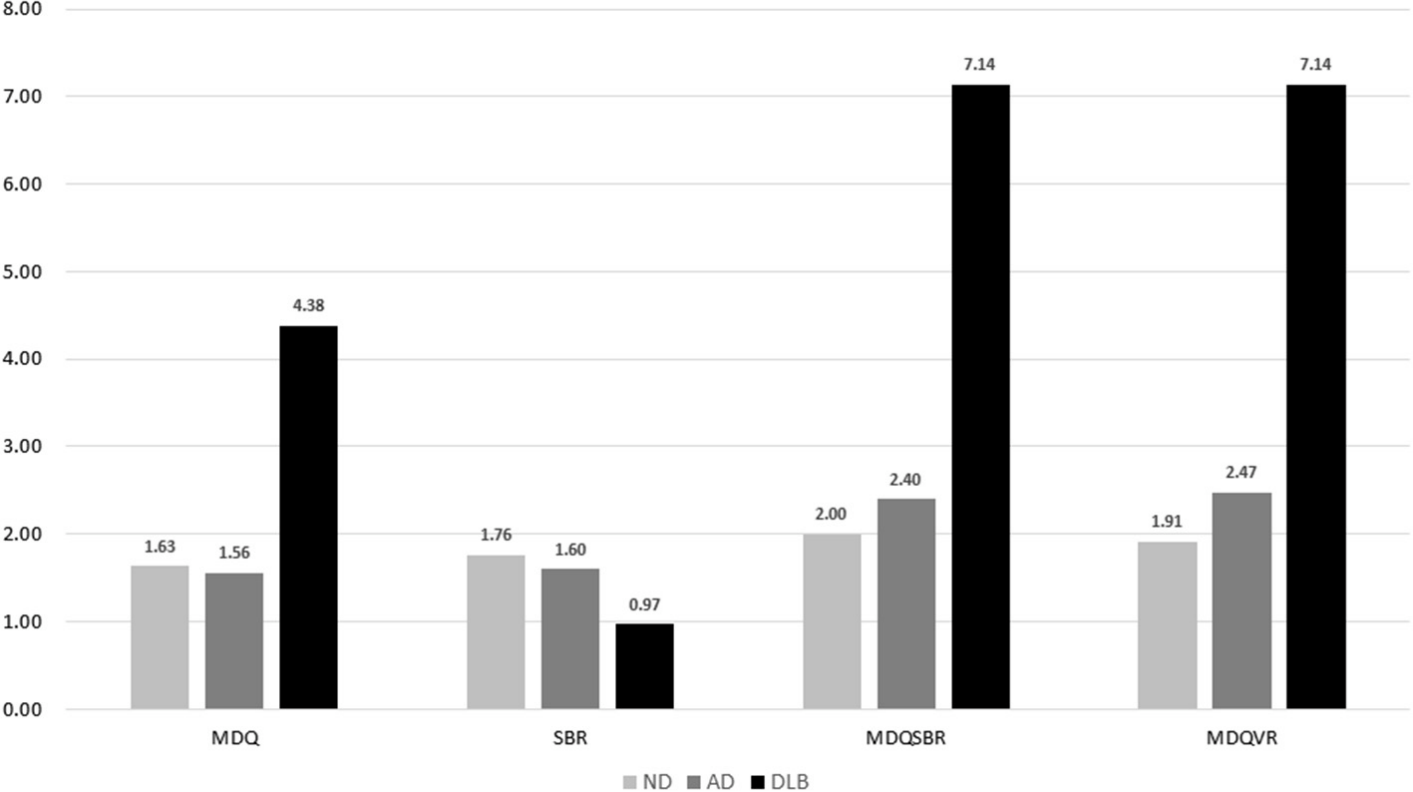
Comparison of MDQ, SBR, MDQSBR, and MDQVR among the ND, AD, and DLB groups. MDQ, motor dysfunction questionnaire in the history-based artificial intelligence clinical dementia diagnostic system; SBR, striatal–background ratio of dopamine transporter imaging; MDQSBR, the composite scale of MDQ and SBR; MDQVR, the composite scale of MDQ and DAT VR scale; ND, non-dementia control; AD, Alzheimer's disease; DLB, dementia with Lewy bodies.

A comparison of the total scores of the four diagnostic tools among the DLB, ND, and AD groups is shown in Figure 2, which shows significantly higher MDQ, MDQVR, and MDQSBR and a significantly lower SBR in the DLB group (all *p* < 0.001).

**Figure 3:**
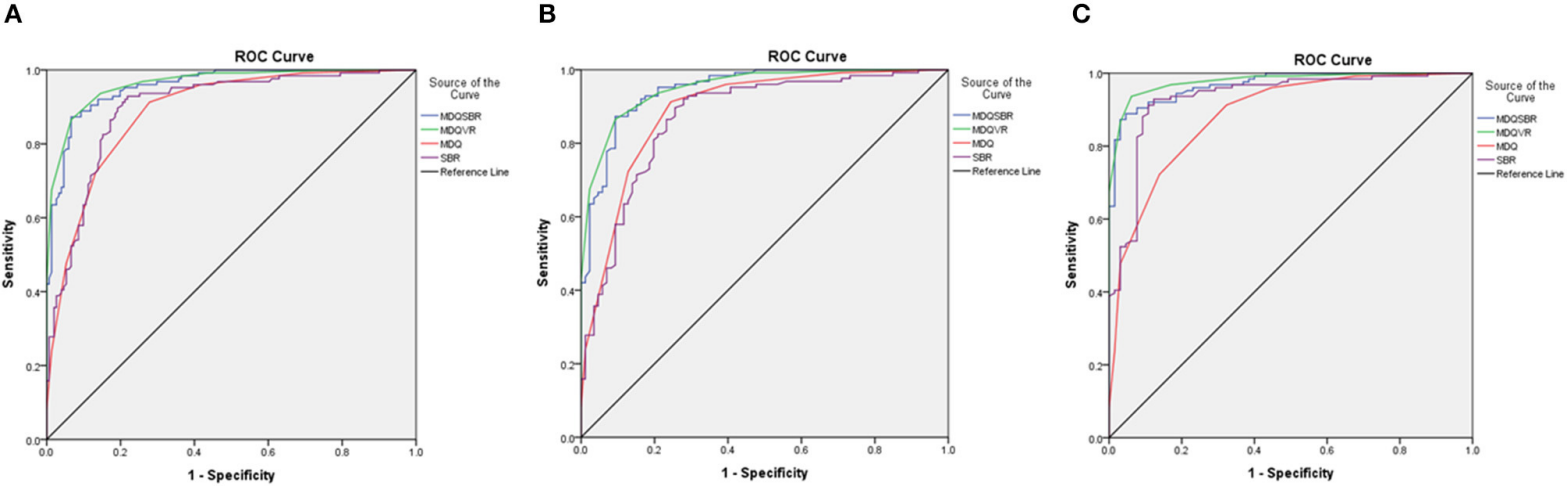
Comparison of ROC curves of MDQ, SBR, MDQSBR composite scale, and MDQVR composite scale among ND, AD, and DLB groups. **(A)** DLB vs. non-DLB (ND+AD). **(B)** DLB vs. AD. **(C)** DLB vs. ND. ROC, receiver operating characteristic; MDQ, motor dysfunction questionnaire in the history-based artificial intelligence clinical dementia diagnostic system; SBR, striatal–background ratio of dopamine transporter imaging; MDQSBR, the composite scale of MDQ and SBR; MDQVR, the composite scale of MDQ and DAT VR scale; ND, non-dementia control; AD, Alzheimer's disease dementia; DLB, dementia with Lewy bodies.

The comparison of receiver operating characteristic (ROC) curves of MDQ, SBR, MDQSBR composite scale, and MDQVR composite scale among the ND, AD, and DLB groups are shown in Figure 3.

To investigate the clinical significance of positive HAI-MDQ among all participants, multivariate risk estimates for all participants in the positive MDQ (HAI-MDQ+) group were compared with the negative (HAI-MDQ–) group with adjustment for age and disease severity (CDR). The HAI-MDQ+ group had a higher diagnosis of PD/DLB (OR = 38.72, *p* < 0.001), lower MoCA (OR = 0.95, *p* = 0.014), lower IADL (OR = 0.69, *p* < 0.001), higher LED (OR = 1.01, *p* = 0.004), higher UPDRS-m (OR = 1.12, *p* < 0.001), lower SBR (OR = 0.07, *p* < 0.001), and higher frequency of all PD/DLB non-motor features, including DATabN (OR = 11.27, *p* < 0.001), RBD (OR = 4.31, *p* < 0.001), cognitive fluctuation (OR = 4.11, *p* < 0.001), and VH (OR = 2.47, *p* = 0.020) (Table 3).

**Table 3:** Multivariate risk estimates (ORs) for all participants in the positive MDQVR composite scale (MDQVR+) group compared with the negative (MDQVR–) group adjusted for age, sex, and CDR-SB.

	**MDQVR+, mean (SD)**	**MDQVR–, mean (SD)**	**ORs**	***p***
N	119	158		
Age, year	76.2 (7.2)	75.8 (9.1)	NA	
CDR-SB	7.5 (4.4)	4.2 (3.8)	NA	
Female, N (%)	90 (54.2)	69 (50.0)	NA	
CASI	48.8 (22.8)	61.3 (22.9)	1.01	NS
MoCA	8.9 (5.9)	12.5 (7.4)	1.01	NS
NPI-sum	14.1 (12.1)	7.9 (9.9)	1.03	0.048
NPI-burden	6.6 (6.0)	3.5 (5.1)	1.06	0.037
UPDRS-m	34.4 (19.4)	17.6 (14.3)	1.06	<0.001
Fluctuation, N (%)	74 (62.2)	29 (18.4)	4.56	<0.001
VH, N (%)	42 (35.3)	21 (13.3)	2.12	0.024
RBD, N (%)	56 (47.1)	31 (19.6)	3.41	<0.001
Parkinsonism	112 (94.1)	68 (43.0)	23.3	<0.001
SBR	0.96 (0.38)	1.64 (0.45)	1.06	<0.001
LED	208.7 (204.5)	69.9 (125.3)	1.01	0.001

*ORs, odds ratio; MDQVR, the composite scale of MDQ and DAT VR scale; CDR-SB, Sum of Boxes of the Clinical Dementia Rating scale; N, number of participants; NA, not applicable; NS, non-significance; CASI, Cognitive Abilities Screening Instrument; MoCA, Montreal Cognitive Assessment; NPI-sum, sum score of Neuropsychiatric Inventory; NPI-burden, caregiver burden score of neuropsychiatric inventory; UPDRS-m, the motor score of the Unified Parkinson's Disease Rating Scale; Fluctuation, fluctuation of cognition; VH, visual hallucinations; RBD, REM sleep behavior disorder; SBR, striatal–background ratio of dopamine transporter imaging; LED, levodopa equivalent dose*.

## Discussion

We retrospectively analyzed the data from a relatively large population with a DAT imaging study along with a complete dementia/motor function survey and obtained some important results. First, after adjustment for age and disease severity by CDR, the participants with DLB in this study revealed significantly more motor dysfunction and higher non-motor features, including fluctuations of cognition, VH, RBD, and DATabN. These findings of the participants with DLB were consistent with the clinical criteria for the diagnosis of DLB (Galvin, 2015; McKeith et al., 2017). A higher frequency of DATabN than that in the non-DLB group (9.2% in ND and 30.2% in AD) was probably because the patients who received DAT imaging in the non-DLB group were clinically considered to have motor dysfunction that needed to be ruled out of the possibility of PD/PDD or DLB. In this study, 25 participants with ET were enrolled and classified into the NC group (28.1%). According to previous findings, DATabN was found in some cases of ET (Isaias et al., 2008; Waln et al., 2015) or AD (Costa et al., 2003; McKeith et al., 2007). Studies on ET showed that patients with ET had higher uptake values compared with those in patients with PD but lower than those in healthy subjects (Isaias et al., 2008; Waln et al., 2015). Studies comparing DLB and AD have also found that DATabN appears in some patients with AD (Costa et al., 2003; McKeith et al., 2007).

Second, instead of the neurological examination by physicians, from the point of view of caregivers, high rates of different manifestations of characteristic motor dysfunction in patients with DLB are noticeable and significantly higher in different stages or subtypes of the disease. In this study, three or more symptoms of HAI-MDQ were reported in 91.2% of patients with DLB, and these were reported to be much lower in NC (32.2%) or AD (24.4%) with motor dysfunction. These results demonstrated much higher characteristic motor symptoms in DLB than those in non-DLB using the HAI-MDQ, which indicated the practical use of the HAI-MDQ for the screening of parkinsonism due to DLB.

In addition, to differentiate DLB from non-DLB using either the HAI-MDQ (sensitivity: 0.91, specificity: 0.72, and AUC: 0.89) or SBR (sensitivity: 0.91, specificity: 0.80, and AUC: 0.90) was satisfied, whereas a combination of both tools (MDQVR) further increased the power of differentiation with a sensitivity of 0.87, a specificity of 0.93, and an AUC of 0.96. Therefore, we are looking forward to combining complex clinical data and biomarkers supplemented with artificial intelligence and deep learning procedures to provide an even better diagnostic tool for the clinical diagnosis of dementia with and without movement disorders.

Third, the factors associated with positive MDQVR in all participants in this study provided clinical evidence of the value of the questionnaire for clinical screening of DLB in non-DLB. Findings of much higher MDQVR total score in the MDQVR+ group (7.5 ± 1.3) than that in the MDQVR– group (2.2 ± 1.8) and higher UPDRS-m subscores in the MDQVR+ group (34.4 ± 19.4) than those in the MDQVR– group (17.6 ± 14.3) indicated a positive correlation of motor dysfunction between the two tools. The correlation coefficient of MDQVR with UPDRS-m is 0.56 in the later analysis. In other words, the DLB motor features can be well-detected and differentiated from non-DLB using a combined scale of both tools. A significantly lower SBR in the MDQVR+ group (1.1 ± 0.5) than that in the MDQVR– group (1.7 ± 0.4) and a high correlation coefficient of MDQVR with SBR (−0.65) indicated a good correlation of the questionnaire with reducing DAT uptake in striatal areas, which is currently the hallmark of brain imaging study for the diagnosis of DLB. Higher rates of non-motor DLB features, including DATabN, RBD, VH, and cognitive fluctuations, were found in the HAI-MDQ+ group, revealing that the MDQ and the composite questionnaire MDQVR for the clinical detection of DLB were simple, practical, and reliable.

This study has several limitations. First, the original HAICDDS questionnaire was written in Chinese. Although we tentatively translated the questionnaire to English, more colloquial and precise translations are required. Second, this study was conducted in only three regional hospitals in Taiwan. Therefore, the findings of different presentations of motor dysfunction might not be generalizable to all patients. Third, the diagnoses of ND, AD, and DLB were based only on clinical criteria. Therefore, the diagnosis of AD was not based on the newest research framework that emphasizes some important biomarkers, including amyloid PET, tau PET, or CSF studies for the diagnosis of AD with or without dementia (Jack et al., 2018). However, detailed clinical information and DAT imaging may help to differentiate DLB from non-DLB, which was supported by robust clinicopathological evidence (Rizzo et al., 2018).

## Conclusion

This study showed that an informant-based motor questionnaire is a practical tool for the screening of characteristic motor symptoms related to DLB, and this should be the first simple clinical questionnaire for the screening of motor dysfunction characteristic of DLB. The diagnostic value of the questionnaire was further confirmed by positive correlations with the DAT imaging study and motor subscores of the UPDRS. Both questionnaires and DAT imaging were effective in differentiating DLB from AD or ND. A combination of both tools can further improve diagnostic accuracy. This simple screening tool can be applied at the bedside and in clinics for the screening of motor dysfunction related to DLB, and it can help non-specialists to detect DLB easily in healthcare settings without neurologists. Embedded in the HAICDDS project, the MDQ diagnosis requires further machine learning techniques using artificial intelligence and is expected to improve the accuracy and efficiency of the clinical diagnosis of DLB and the differential diagnosis of AD from DLB. Further study of the HAI-MDQ on the discrimination or detection of parkinsonism due to PD and PDD is warranted and is currently in progress.

## Data Availability Statement

The original contributions presented in the study are included in the article/Supplementary Material, further inquiries can be directed to the corresponding authors.

## Ethics Statement

The studies involving human participants were reviewed and approved by the institutional review board of Show Chwan Memorial Hospital. Written informed consent for participation was not required for this study in accordance with the national legislation and the institutional requirements.

## Author Contributions

P-YC undertook the literature search and data analysis and was mainly responsible for revisions and drafts of the manuscript. S-LW contributed to revisions and the final draft of the manuscript. G-UH undertook the literature search and contributed to revisions. C-YW contributed to revisions of the manuscript. All authors contributed to the article and approved the submitted version.

## Conflict of Interest

The authors declare that the research was conducted in the absence of any commercial or financial relationships that could be construed as a potential conflict of interest.

## Publisher's Note

All claims expressed in this article are solely those of the authors and do not necessarily represent those of their affiliated organizations, or those of the publisher, the editors and the reviewers. Any product that may be evaluated in this article, or claim that may be made by its manufacturer, is not guaranteed or endorsed by the publisher.

## References

[B1] Ballard C. McKeith I. Burn D. Harrison R. O'Brien J. Lowery K.. (1997). The UPDRS scale as a means of identifying extrapyramidal signs in patients suffering from dementia with Lewy bodies. Acta Neurol. Scand. 96, 366–371. 10.1111/j.1600-0404.1997.tb00299.x 9449473

[B2] Chan D. K. Hung W. T. Wong A. Hu E. Beran R. G. (2000). Validating a screening questionnaire for parkinsonism in Australia. J. Neurol. Neurosurg. Psychiatry 69, 117–120. 10.1136/jnnp.69.1.117 10864617 PMC1736988

[B3] Chang S. F. Su C. L. Chen Z. Y. Lee C. S. Chen R. C. (1996). Neuroepidemiological survey in Ilan, Taiwan (NESIT)(1): validation of screening instrument in an out-patient department population. Acta Neurol. Taiwan 5, 105–110.

[B4] Chen K. L. Xu Y. Chu A. Q. Ding D. Liang X. N. Nasreddine Z. S.. (2016). Validation of the Chinese version of montreal cognitive assessment basic for screening mild cognitive impairment. J. Am. Geriatr. Soc. 64, e285–e290. 10.1111/jgs.14530 27996103

[B5] Chiu P. Y. Tang H. Wei C. Y. Zhang C. Hung G. U. Zhou W. (2019a). NMD-12: a new machine-learning derived screening instrument to detect mild cognitive impairment and dementia. PLoS ONE 14:e0213430. 10.1371/journal.pone.0213430 30849106 PMC6407752

[B6] Chiu P. Y. Wei C. Y. Hung G. U. (2019b). Preliminary study of the history-based artificial intelligent clinical dementia diagnostic system. Show Chwan Med. J. 18, 18–27. 10.3966/156104972019061801003

[B7] Costa D. C. Walker Z. Walker R. W. Fontes F. R. (2003). Dementia with Lewy bodies versus Alzheimer's disease: role of dopamine transporter imaging. Mov. Disord. 18(Suppl. 7), S34–S38. 10.1002/mds.10576 14531044

[B8] Dahodwala N. Siderowf A. Baumgarten M. Abrams A. Karlawish J. (2012). Screening questionnaires for parkinsonism: a systematic review. Parkinsonism Relat. Disord. 18, 216–224. 10.1016/j.parkreldis.2011.09.003 21930414 PMC3253331

[B9] Emre M. Aarsland D. Brown R. Burn D. J. Duyckaerts C. Mizuno Y.. (2007). Clinical diagnostic criteria for dementia associated with Parkinson's disease. Mov. Disord. 22, 1689–1707. 10.1002/mds.21507 17542011

[B10] Galvin J. E. (2015). Improving the clinical detection of Lewy body dementia with the Lewy body composite risk score. Alzheimers Dement. (Amst) 1, 316–324. 10.1016/j.dadm.2015.05.004 26405688 PMC4576496

[B11] Isaias I. U. Canesi M. Benti R. Gerundini P. Cilia R. Pezzoli G.. (2008). Striatal dopamine transporter abnormalities in patients with essential tremor. Nucl. Med. Commun. 29, 349–353. 10.1097/MNM.0b013e3282f4d307 18317299

[B12] Jack Jr C. R Bennett D. A. Blennow K. Carrillo M. C. Dunn B. Haeberlein S. B.. (2018). NIA-AA Research Framework: Toward a biological definition of Alzheimer's disease. Alzheimers Dement. 14, 535–562. 10.1016/j.jalz.2018.02.018 29653606 PMC5958625

[B13] Lawton M. P. Brody E. M. (1969). Assessment of older people: self-maintaining and instrumental activities of daily living. Gerontologist 9, 179–186. 10.1093/geront/9.3_Part_1.179 5349366

[B14] Lin C. M. Hung G. U. Wei C. Y. Tzeng R. C. Chiu P. Y. (2018). An informant-based simple questionnaire for language assessment in neurodegenerative disorders. Dement. Geriatr. Cogn. Disord. 46, 207–216. 10.1159/000493540 30336484

[B15] Lopez O. L. Wisnieski S. R. Becker J. T. Boller F. DeKosky S. T. (1997). Extrapyramidal signs in patients with probable Alzheimer disease. Arch. Neurol. 54, 969–975. 10.1001/archneur.1997.00550200033007 9267971

[B16] McKeith I. O'Brien J. Walker Z. Tatsch K. Booij J. Darcourt J.. (2007). Sensitivity and specificity of dopamine transporter imaging with 123I-FP-CIT SPECT in dementia with Lewy bodies: a phase III, multicentre study. Lancet Neurol. 6, 305–313. 10.1016/S1474-4422(07)70057-1 17362834

[B17] McKeith I. G. Boeve B. F. Dickson D. W. Halliday G. Taylor J. P. Weintraub D.. (2017). Diagnosis and management of dementia with Lewy bodies: fourth consensus report of the DLB Consortium. Neurology 89, 88–100. 10.1212/WNL.0000000000004058 28592453 PMC5496518

[B18] McKeith I. G. Dickson D. W. Lowe J. Emre M. O'Brien J. T. Feldman H.. (2005). Diagnosis and management of dementia with Lewy bodies: third report of the DLB Consortium. Neurology 65, 1863–1872. 10.1212/01.wnl.0000187889.17253.b1 16237129

[B19] McKhann G. M. Knopman D. S. Chertkow H. Hyman B. T. Jack C. R. Jr. Kawas C. H.. (2011). The diagnosis of dementia due to Alzheimer's disease: recommendations from the National Institute on Aging-Alzheimer's Association workgroups on diagnostic guidelines for Alzheimer's disease. Alzheimers Dement. 7, 263–269. 10.1016/j.jalz.2011.03.005 21514250 PMC3312024

[B20] Mutch W. J. Smith W. C. Scott R. F. (1991). A screening and alerting questionnaire for parkinsonism. Neuroepidemiology 10, 150–156. 10.1159/000110261 1922649

[B21] Nihashi T. Ito K. Terasawa T. (2020). Diagnostic accuracy of DAT-SPECT and MIBG scintigraphy for dementia with Lewy bodies: an updated systematic review and Bayesian latent class model meta-analysis. Eur. J. Nucl. Med. Mol. Imaging. 47, 1984–1997. 10.1007/s00259-019-04480-8 31423561

[B22] Postuma R. B. Berg D. Stern M. Poewe W. Olanow C. W. Oertel W.. (2015). MDS clinical diagnostic criteria for Parkinson's disease. Mov. Disord. 30, 1591–1601. 10.1002/mds.26424 26474316

[B23] Rizzo G. Arcuti S. Copetti M. Alessandria M. Savica R. Fontana A.. (2018). Accuracy of clinical diagnosis of dementia with Lewy bodies: a systematic review and meta-analysis. J. Neurol. Neurosurg. Psychiatry 89, 358–366. 10.1136/jnnp-2017-316844 29030419

[B24] Sasaki S. (2018). High prevalence of parkinsonism in patients with MCI or mild Alzheimer's disease. Alzheimers Dement. 14, 1615–1622. 10.1016/j.jalz.2018.06.3054 30222946

[B25] Tanner C. M. Gilley D. W. Goetz C. G. (1990). A brief screening questionnaire for parkinsonism. Ann. Neurol. 28, 267–268.

[B26] The Italian Longitudinal Study on Aging Working Group. (1997). Prevalence of chronic diseases in older Italians: comparing self-reported and clinical diagnoses. The Italian Longitudinal Study on Aging Working Group. Int. J. Epidemiol. 26, 995–1002. 10.1093/ije/26.5.995 9363520

[B27] Waln O. Wu Y. Perlman R. Wendt J. Van A. K. Jankovic J. (2015). Dopamine transporter imaging in essential tremor with and without parkinsonian features. J. Neural Transm. (Vienna) 122, 1515–1521. 10.1007/s00702-015-1419-z 26133163

[B28] Wang C. T. Hung G. U. Wei C. Y. Tzeng R. C. Chiu P. Y. (2020). An informant-based simple questionnaire for visuospatial dysfunction assessment in dementia. Front. Neurosci. 14:44. 10.3389/fnins.2020.00044 32082114 PMC7006475

[B29] Zaccai J. McCracken C. Brayne C. (2005). A systematic review of prevalence and incidence studies of dementia with Lewy bodies. Age Ageing 34, 561–566. 10.1093/ageing/afi190 16267179

[B30] Zhu F. Li X. McGonigle D. Tang H. He Z. Zhang C.. (2020). Analyze informant-based questionnaire for the early diagnosis of senile dementia using deep learning. IEEE J. Transl. Eng. Health Med. 8:2200106. 10.1109/JTEHM.2019.2959331 31966933 PMC6964964

