# Supplementary material for: Motor Dysfunction Questionnaire and Dopamine Transporter Imaging Composite Scale Improve Differentiating Dementia With Lewy Bodies From Alzheimer's Disease With Motor Dysfunction
Source: Front Aging Neurosci. 2021 Aug 11;13:709215. doi: 10.3389/fnagi.2021.709215 (PMC8385672; doi:10.3389/fnagi.2021.709215)
Supplement: Supplementary file 1 [file Table_1.DOCX]

Supplementary Material

**Supplementary Table 1.** Composition of the HAICDDS-Motor Dysfunction Questionnaire (HAI-MDQ). * selected items.

| Item |  | PD/DLB  **(n = 253) % yes** | Non-PD/DLB  **(n = 491) % yes** | *X^2^* |
| --- | --- | --- | --- | --- |
| MD01* | 坐著或靜止不動的時候，肢體或臉部會明顯地顫抖嗎? | 48.2 | 7.5 | 164.5 |
|  | Does tremor, or shaking, often in hands, arms, or legs, occur when he/she is sitting or standing still? |  |  |  |
| MD02* | 拿東西，做動作或是說話的時候，肢體或臉部會明顯地顫抖嗎? | 54.5 | 15.5 | 124.4 |
|  | Does tremor, or shaking, often in hands, arms, or legs, occur when he/she is reaching something, holding position, or talking? |  |  |  |
| MD03* | 動作變得緩慢，尤其是起步特別困難，面部表情也明顯減少嗎? | 40.7 | 16.3 | 53.7 |
|  | Does movement become slower when he/she try to move from a resting position, and also decreases facial expression? |  |  |  |
| MD04* | 動作變得僵硬，手腳彎曲的角度受到限制，走路步伐變小身體會前傾嗎? | 85.0 | 64.4 | 34.8 |
|  | Does limited angle or stiff movement occur during moving, the steps become smaller and the posture become stooped? |  |  |  |
| MD05* | 動作變得不穩，走路不平衡，有時候好像要跌倒或真的跌倒嗎? | 56.9 | 26.7 | 65.5 |
|  | Does balance or posture problems occur during walking and cause frequent falls? |  |  |  |
| MD06 | 是否常常坐立不安？ | 25.3 | 15.3 | 11.0 |
|  | Does he/she feel restless? |  |  |  |
| MD07* | 若有動作障礙，剛開始的時候就常常跌倒? | 33.2 | 14.9 | 33.7 |
|  | Did he/she fall often at the beginning if he/she has these motor dysfunctions mentioned above? |  |  |  |
| MD08* | 說話音調變得平淡，比較沒有抑揚頓挫，會越講越小聲嗎? | 32.1 | 2.1 | 45.6 |
|  | Does the speech reduce pitch range (monotone) and volume (hypophonia)? |  |  |  |
| MD09 | 走路或駕車是否常常偏向一邊? | 26.1 | 11.4 | 26.3 |
|  | Does he/she often deviate to one side during walking, riding, or driving on the road? |  |  |  |
| MD10 | 手腳或身體會不自主跳動嗎? | 8.3 | 2.0 | 16.4 |
|  | Does jerking movement occur in hands, arms, legs, or trunk? |  |  |  |
| MD11 | 是否有持續扭轉的姿勢或體位嗎? | 2.8 | 1.5 | 1.4 |
|  | Does posture or movement become repetitive or twisting? |  |  |  |
| MD12 | 動作障礙剛開始的時候是否只影響一邊的肢體？ | 40.5 | 20.7 | 13.8 |
|  | Did the motor dysfunction start in one side of the face, hand, or leg? |  |  |  |
| MD13 | 是否作精細動作有顯著困難？ | 50.6 | 28.7 | 24.6 |
|  | Did fine motor movement become harder and harder? |  |  |  |
